# Establishment of a novel glycolysis-related prognostic gene signature for ovarian cancer and its relationships with immune infiltration of the tumor microenvironment

**DOI:** 10.1186/s12967-021-03057-0

**Published:** 2021-09-08

**Authors:** Jianlei Bi, Fangfang Bi, Xue Pan, Qing Yang

**Affiliations:** 1 Department of Obstetrics and Gynecology, Shengjing Hospital of China Medical University, Shenyang, China; 2 Department of Obstetrics and Gynecology, The Second Hospital of Dalian Medical University, Dalian, China

**Keywords:** Ovarian cancer, Glycolysis, Tumor microenvironment, Immune alterations, Prognostic signature

## Abstract

**Background:**

Glycolysis affects tumor growth, invasion, chemotherapy resistance, and the tumor microenvironment. In this study, we aimed to construct a glycolysis-related prognostic model for ovarian cancer and analyze its relationship with the tumor microenvironment’s immune cell infiltration.

**Methods:**

We obtained six glycolysis-related gene sets for gene set enrichment analysis (GSEA). Ovarian cancer data from The Cancer Genome Atlas (TCGA) database and two Gene Expression Omnibus (GEO) datasets were divided into two groups after removing batch effects. We compared the tumor environments' immune components in high-risk and low-risk groups and analyzed the correlation between glycolysis- and immune-related genes. Then, we generated and validated a predictive model for the prognosis of ovarian cancer using the glycolysis-related genes.

**Results:**

Overall, 27/329 glycolytic genes were associated with survival in ovarian cancer, 8 of which showed predictive value. The tumor cell components in the tumor microenvironment did not differ between the high-risk and low-risk groups; however, the immune score differed significantly between groups. In total, 13/24 immune cell types differed between groups, including 10 T cell types and three other immune cell types. Eight glycolysis-related prognostic genes were related to the expression of multiple immune-related genes at varying degrees, suggesting a relationship between glycolysis and immune response.

**Conclusions:**

We identified eight glycolysis-related prognostic genes that effectively predicted survival in ovarian cancer. To a certain extent, the newly identified gene signature was related to the tumor microenvironment, especially immune cell infiltration and immune-related gene expression. These findings provide potential biomarkers and therapeutic targets for ovarian cancer.

**Supplementary Information:**

The online version contains supplementary material available at 10.1186/s12967-021-03057-0.

## Background

Ovarian cancer is one of the most common malignant tumors of the female reproductive system. Because there is a void of information about the incidence of ovarian cancer and insufficient early detection methods, approximately 60–70% of diagnoses happen after the tumor has grown to an advanced stage (International Federation of Gynecology and Obstetrics stage III/IV) [1]. Thus, the mortality rate due to ovarian cancer ranks first among gynecological malignancies, presenting a severe threat to women’s health. Despite significant progress in the treatment options and availability of therapeutics, especially with regard to emerging immunotherapies, the prognosis of ovarian cancer remains poor; approximately 80% of patients with advanced ovarian cancer will enter remission and eventually succumb to the disease. Further, the histological grade and stage of the tumor determine the prognosis of ovarian cancer; however, patients with tumors of similar stages may have different forecasts. Therefore, an urgent need exists to investigate the pathogenic mechanisms underlying ovarian cancer and establish a reliable predictive model for the prognosis of patients diagnosed with ovarian cancer.

The deregulation of cellular energetics or metabolic reprogramming is a key characteristic of tumor cells, closely related to tumor occurrence, progression, and drug resistance [2]. Normal cells rely on glycolysis, instead of oxygen-consuming β-oxidation, to provide energy during hypoxia. However, in tumor cells, glycolysis tends to occur even in aerobic environments. Warburg [3] first reported this phenomenon and termed it the “Warburg effect” or “aerobic glycolysis.” Although ATP production per molecule of glucose through glycolysis is low, the ATP yield is much faster than oxidative phosphorylation and can meet cancer cells’ demands during rapid growth and proliferation. In many tumors, glucose metabolism reprogramming has been verified, and increased glycolysis reportedly promotes biomass biosynthesis [4].

Some glycolysis-related molecules are also closely related to tumor proliferation, invasion, and autophagy. For example, solute carrier family 2 member 1 (SLC2A1), also called GLUT1, a glucose transporter involved in the first step of glycolysis, is a direct target of miR-22 in breast cancer, and miR-22 dysregulation can inhibit cell proliferation and invasion via GLUT1 [5]. GLUT1 is a potential prognostic marker for colorectal cancer, with a predictive value for survival rates after liver metastasis resection [6]. Aldolase catalyzes the conversion of fructose-1,6-diphosphate to glyceraldehyde-3-phosphate and dihydroxyacetone phosphate in glycolysis. High levels of aldolase A in lung squamous cell carcinoma are related to various clinical parameters, including metastasis, grade, differentiation, survival rate, and prognosis [7].

Accumulating evidence indicates a relationship between glycolysis and the tumor microenvironment or immune evasion [8]. On the one hand, activated immune cells can induce glycolysis, similar to that of tumor cells. For instance, the proliferation of activated T cells is often glycolysis dependent [9]. On the other hand, if cancer cells primarily utilize glucose to fuel glycolysis, competition between cancer cells and immune cells for glucose is likely to limit the immune activity in tumor microenvironment.

Little is known regarding glycolysis-related genes with a predictive value and the relationship between glycolysis and immune cell infiltration in ovarian cancer. In this study, we aimed to construct a glycolysis-related prognostic model for ovarian cancer and analyze its relationship with tumor microenvironment and immune cell infiltration. We obtained ovarian cancer datasets from TCGA and GEO and randomly divided data into training and test sets. Eight glycolytic genes related to prognosis were identified from the training set and verified in the test set.

## Materials and methods

### Data collection and preprocessing

The RNA-sequence profiles and corresponding clinical data of 581 patients with ovarian cancer were downloaded from TCGA (https://portal.gdc.cancer.gov/) (n = 364) and GEO (https://www.ncbi.nlm.nih.gov/geo/) (GSE17260, n = 110; GSE73614, n = 107). Both GEO datasets were based on the GPL6480 platform (Agilent-014850 Whole Human Genome Microarray 4 × 44 K G4112F). The ovarian cancer samples downloaded from the TCGA and GEO databases in this study are of primary ovarian tumors and do not contain borderline tumors. To eliminate differences between batches, we used the “sva” package in R software for normalization. We obtained 16,889 common genes for subsequent analyses. Additional file 2: Table S1 shows the clinical characteristics of 581 patients with ovarian cancer. We randomly assigned the cohort into a training set (n = 292), which we used to build our predictive model, and a test set (n = 289) to verify the model.

### Glycolysis-related gene sets

Molecular Signatures Database (MSigDB, http://www.broad.mit.edu/gsea/msigdb/) is a collection of annotated gene sets for GSEA. Six glycolysis-related gene sets were extracted, including REACTOME_GLYCOLYSIS, KEGG_GLYCOLYSIS_GLUCONEOGENESIS, GO_GLYCOLYTIC_PROCESS, BIOCARTA_GLYCOLYSIS_PATHWAY, HALLMARK_GLYCOLYSIS, and BIOCARTA_FEEDER_PATHWAY. We identified 329 glycolysis-related genes from 581 patients using the “limma” package in R software.

### Glycolysis-related prognostic gene signatures

We performed Univariate Cox regression analysis on the training set to identify genes significantly related to the overall survival time using the “survival” package in R software; a p-value < 0.05 indicated statistically significance. We used lasso regression analysis to prevent model overfitting. Subsequently, we determined the risk gene signatures were using multivariate Cox analysis.

### Analyses of prognosis

Based on gene expression levels (expr) and regression coefficients (coef) in the multivariate Cox regression analysis for the training cohort, a glycolytic risk model was constructed as follows:

$${\text{Risk Score}}\, = \,{\text{expr}}\_{\text{gene}}\_{1}\, \times \,{\text{coef}}\_{\text{gene}}\_{1}\, + \,{\text{expr}}\_{\text{gene}}\_{2}\, \times \,{\text{coef}}\_{\text{gene}}\_{2}\, + \, \ldots \, + \,{\text{expr}}\_{\text{gene}}\_{\text{n}}\, \times \,{\text{coef}}\_{\text{gene}}\_{\text{n}}.$$

Our group then used this formula to calculate the risk score for each patient. We divided patients into high-risk and low-risk groups by using the median risk score of 1.03565 as the cut-off value,

We generated a heatmap of the glycolysis-related prognostic signatures and survival curves using the “pheatmap” and “survival” packages in R software, respectively. Then, we performed univariate and multivariate analyses on the histological grade, clinical stage, and risk score data. Receiver operating characteristic (ROC) curves were drawn to verify the model’s predictive validity, and the area under the curve (AUC) was calculated with the training and test sets using the “survivalROC” package in R software.

### Mutation analyses of glycolysis-related prognostic signatures

cBioPortal (http://www.cbioportal.org/) is a database that integrates various genomic data types, including somatic mutations, DNA copy number alterations, mRNA and miRNA expression levels, DNA methylation, protein abundance, and phosphoprotein abundance. We queried the genes identified in the analysis of TCGA datasets against the cBioPortal database to explore the prognostic signatures’ genomic characteristics.

### Association between the tumor microenvironment and glycolytic risk model

Cellular components in the tumor microenvironment consist of cancer cells, immune cells, and stromal cells. These cells form a continually evolving microenvironment by secreting specific molecules and expressing a wide range of receptors. We calculated the stromal cell score and immune cell score for 581 samples using the “estimate” package in R software. A higher score indicated a higher component frequency in the sample. The estimated score (the sum of the stromal cell and immune cell scores) is indicative of the abundance of cancer cell components in the sample. We evaluated tumor microenvironment differences between the high- and low-risk groups based on our risk model using t-tests. We also analyzed the relationship between the prognostic signature and tumor microenvironment.

### Relationships between immune cell profiles and the glycolytic risk model

Immune cell abundance identifier (ImmuCellAI, http://bioinfo.life.hust.edu.cn/ImmuCellAI#!/) is a web tool for the quantitative evaluation of 24 immune cells, including 18 T-cell subtypes, B cells, natural killer (NK) cells, monocyte cells, macrophage cells, neutrophils, and dendritic cells (DCs). We used this tool to evaluate the RNA-sequencing profiles for 581 cases and determine each sample's immune cell components. Differences between the high-risk and low-risk groups were assessed, and correlations between each immune cell type and the prognostic signature were analyzed.

Further, an immune-related gene list was extracted from ImmPort (https://www.immport.org/home). The expression levels of glycolysis-related prognostic genes and immune-related genes in the complete set of 581 cases were analyzed. An interaction network of immune-related genes, immune cells, and prognostic signatures was generated using Cytoscape (version 3.8.2).

### Statistical analysis

R software (version 4.0.3) was used for statistical analyses and the visualization of results. A *p*-value < 0.05 was considered to indicate statistical significance. Correlation coefficients with an absolute value greater than 0.2 and *p* < 0.05 were deemed to be significant.

## Results

### Identification of eight glycolysis-related prognostic genes using the training set

Based on the univariate analysis of the training set, 27 genes were related to ovarian cancer prognosis (Table 1). After lasso regression (Additional file 1: Fig. S1) and multivariate analyses, we obtained a prognostic signature composed of up to eight of the following genes: actinin alpha 3 (*ACTN3*), artemin (*ARTN*), CXC motif chemokine receptor 4 (*CXCR4*), decorin (*DCN*), estrogen-related receptor beta (*ESRRB*), fructose-bisphosphatase 1 (*FBP1*), GDP-mannose pyrophosphorylase B (*GMPPB*), and proteasome 26S subunit, ATPase 4 (*PSMC4*). Among these, *CXCR4*, *FBP1*, *ARTN*, and *GMPPB* correlated with favorable prognoses, whereas *ACTN3*, *ESRRB*, *DCN*, and *PSMC4* were associated with poor prognoses (Table 1).

**Table 1:** Univariable and multivariable Cox analyses of glycolysis-related genes in ovarian cancer

Gene	Univariable Cox analysis				Multivariable Cox analysis				
	HR	HR.95L	HR.95H	p-value	Coef.	HR	HR.95L	HR.95H	p-value
GMPPB	0.835	0.730	0.954	0.008	− 0.149	0.862	0.754	0.984	0.028
ARTN	0.847	0.761	0.943	0.002	− 0.109	0.896	0.800	1.005	0.061
FBP1	0.965	0.944	0.987	0.002	− 0.0356	0.965	0.942	0.989	0.004
CXCR4	0.989	0.982	0.996	0.002	− 0.011	0.989	0.982	0.996	0.002
PSMC4	1.003	1.001	1.006	0.004	0.002	1.002	1.000	1.004	0.086
DCN	1.011	1.003	1.019	0.010	0.008	1.008	1.000	1.017	0.049
ESRRB	1.359	1.094	1.688	0.006	0.341	1.407	1.109	1.784	0.005
ACTN3	1.819	1.305	2.537	0.000	0.356	1.427	0.950	2.144	0.087
LDHB	1.003	1.001	1.005	0.007					
CD44	0.955	0.921	0.989	0.010					
TPST1	1.080	1.018	1.146	0.011					
ALDH3B2	0.983	0.970	0.996	0.013					
PYGB	1.025	1.005	1.046	0.014					
ARNT	1.054	1.011	1.099	0.014					
SLC37A4	0.921	0.861	0.985	0.017					
B3GALT6	0.956	0.920	0.993	0.020					
PGM2L1	0.904	0.827	0.987	0.024					
TGFBI	1.011	1.001	1.021	0.030					
ISG20	0.889	0.798	0.990	0.032					
STC2	0.933	0.876	0.994	0.033					
EGFR	1.077	1.006	1.153	0.034					
VCAN	1.028	1.002	1.054	0.035					
PRKAG3	1.582	1.031	2.428	0.036					
NUP107	1.027	1.002	1.053	0.037					
NSDHL	0.970	0.943	0.998	0.039					
HS2ST1	0.891	0.798	0.994	0.039					
TFF3	0.989	0.978	1.000	0.049					

**Fig. 1:**
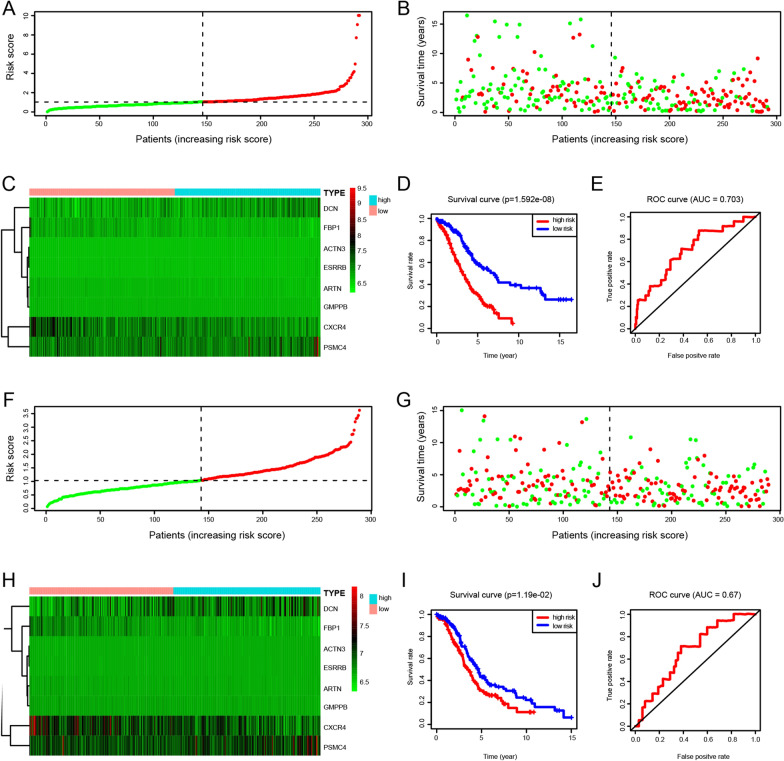
Establishment and verification of an eight glycolysis-related gene-based prognostic signatures in ovarian cancer. **A** Risk score distribution in patients classified as low-risk (n = 146) and high-risk (n = 146) in the training set. **B** Survival status of low-risk and high-risk patients in the training set. **C** Heatmap of eight glycolysis-related risk gene expression levels in the training set. **D** Kaplan–Meier curves for the high-risk and low-risk groups in the training set. **E** ROC analysis of the glycolytic risk model using the training set. **F** Risk score distribution for low-risk (n = 143) and high-risk (n = 146) patients in the test set. **G** Survival status for the high-risk and low-risk groups in the test set. **H** Heatmap of eight glycolysis-related risk gene expression levels in the test set. **I** Kaplan–Meier curves for the high-risk and low-risk groups in the test set. **J** ROC analysis of the glycolytic risk model using the testing set

Based on coefficients for the eight prognostic genes, the following risk model was established:

Risk score = (0.356 × *ACTN3*) + (0.341 × *ESRRB*) + (0.008 × DCN) + (0.002 × *PSMC4*) – (0.011 × *CXCR4*) – (0.036 × *FBP1*) – (0.109 × *ARTN*) – (0.149 × *GMPPB*). The risk score for each patient was calculated. Patients in the training set were divided into high-risk (n = 146) and low-risk (n = 146) groups (Fig. 1A) using a risk score of 1.03565 as a cut-off. Patients’ survival status and prognostic signatures are summarized in Fig. 1B, C. Kaplan–Meier survival analysis indicated a significant difference in survival rates between the high-risk and low-risk groups (*p* < 0.05) (Fig. 1D). The AUC of the risk model for the training set was 0.703 (Fig. 1E).

### Validation of the risk model based on the eight glycolysis-related genes using the test set

To test the robustness of the predictive value for the prognostic gene signature, we repeated the analyses described above using the test set. Of the 289 patients in the test set, 143 were assigned to the low-risk group and 146 to the high-risk group based on the same median risk score of the training set (Fig. 1F). The survival status and heatmaps for the eight prognostic genes in the test set are summarized in Fig. 1G, H. In the survival analysis, the prognosis was worse in the high-risk group than in the low-risk group, similar to the results obtained using the training set (Fig. 1I). The AUC value was 0.67, indicating that the risk model had good predictive value (Fig. 1J).

### Analyses of prognosis and the predictive accuracy of the prognostic gene signature based on the eight glycolysis-related genes

**Fig. 2:**
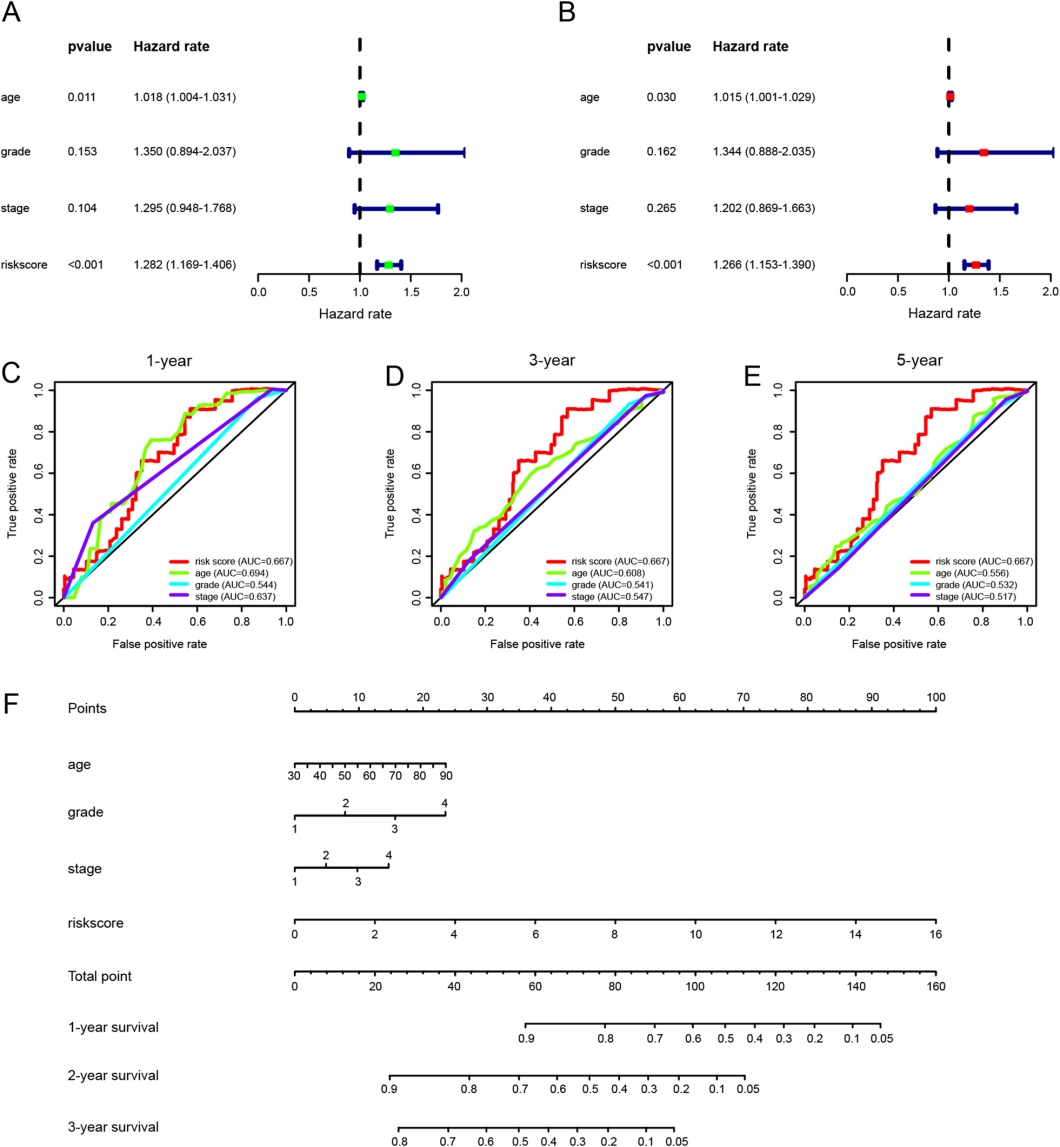
Predictive accuracy of the eight glycolysis-related gene-based prognostic signature. **A** Univariate Cox analysis of risk scores and clinical features associated with overall survival in the dataset from TCGA. **B** Multivariate Cox analysis of risk scores and clinical features associated with overall survival in the dataset from TCGA. **C** ROC analyses of the specificity and sensitivity of the risk score, age, pathological grade, and clinical stage in predicting prognosis in ovarian cancer. **D** Nomogram for predicting the 1-, 2-, and 3-year survival rates in ovarian cancer

Using the dataset obtained from TCGA, we analyzed correlations between prognosis and clinical features, including age, pathological grade, and clinical stage. The hazard ratios (HRs) for patient age, pathological grade, and clinical stage returned risk scores of 1.018, 1.350, 1.295, and 1.282, respectively, after univariate Cox analysis (Fig. 2A). In multivariate Cox analysis, the HRs for age, pathological grade, clinical stage, and risk score were 1.015, 1.344, 1.202, and 1.266, respectively (Fig. 2B). In both univariate and multivariate Cox analyses, the glycolytic risk model was an independent prognostic indicator (*p* < 0.05).

A ROC analysis was performed to assess the specificity and sensitivity of the risk score and clinical features to predict prognosis. As shown in Fig. 2C, the AUC for the risk score was better than that for other clinical features at 1, 3, and 5 years, indicating that the risk model independently predicted the survival rate in ovarian cancer.

A nomogram was constructed by integrating the risk score and clinical features, including age, pathological grade, and clinical stage. The nomogram was used to evaluate each variable score, and by calculating the total score, we estimated the 1-year, 3-year, and 5-year survival rates (Fig. 2D).

### Alterations of the eight glycolysis-related prognostic genes

**Fig. 3:**
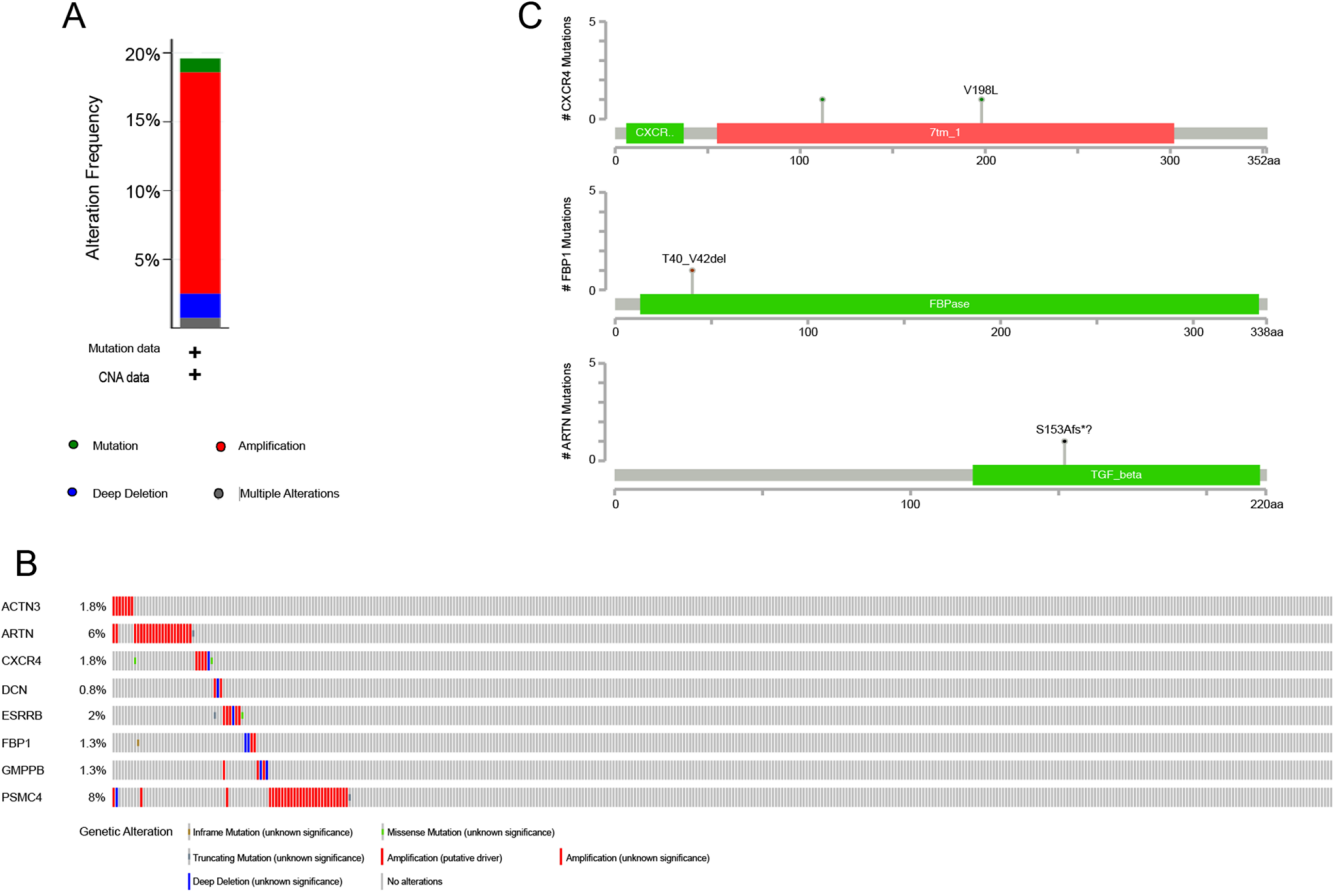
Alterations in the eight glycolysis-related prognostic genes. **A** Alteration frequencies in 398 patients with ovarian cancer in the cBioPortal database. **B** Alteration frequencies for the eight risk genes for ovarian cancer in the cBioPortal database. **C** Mutations in *GMPPB*, *CXCR4*, and *ARTN* in ovarian cancer cases in the cBioPortal database

We analyzed mutations in the eight prognostic genes using the cBioPortal database. The mutation frequencies of these genes in ovarian cancer cases were not high (Fig. 3A). In particular, alterations in the eight prognostic genes were detected in 78 of 398 patients. The types of alterations included in-frame, missense, nonsense mutations, amplifications, and deep deletions, of which amplifications were the dominant type. Among the eight prognostic genes, *PMSC4* had the highest alteration frequency (i.e., 8%), followed by *ARTN* (6%) (Fig. 3B). Mutations in *GMPPB*, *CXCR4*, and *ARTN* occurred in protein domains; although they may affect gene function, these mutations were rare (Fig. 3C). These results indicate that the sequences of glycolytic genes are relatively conserved in ovarian cancer.

### Relationships between the glycolytic risk model and the tumor microenvironment

**Fig. 4:**
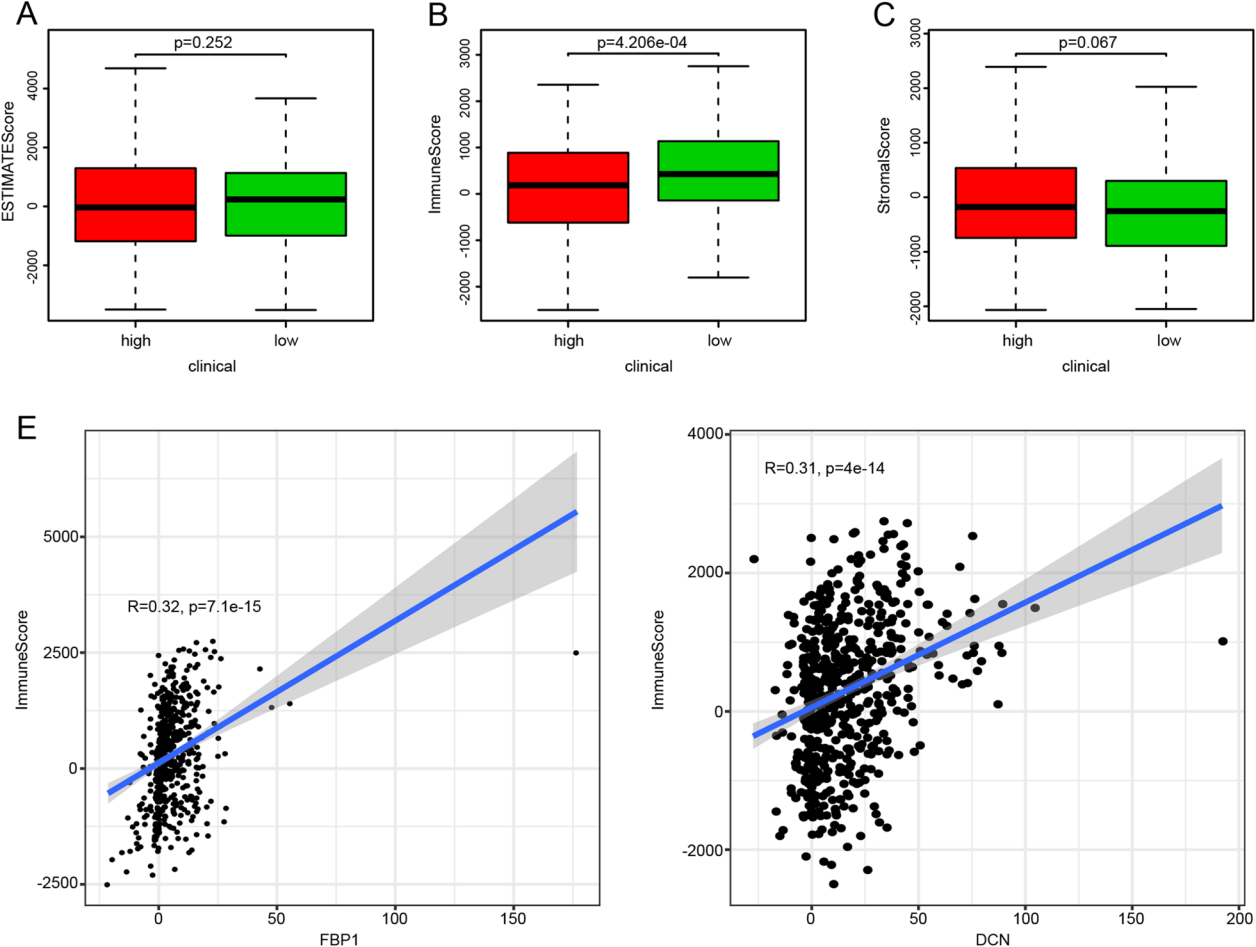
Tumor microenvironment in the high-risk group and the low-risk group for the entire cohort. **A** Stromal score. **B** Immune score. **C** Estimate score. **D** Correlations between levels of *DCN* and *FBP1* and immune scores (*p* < 0.05)

The tumor microenvironments of the entire cohort were analyzed. The stromal score was slightly higher in the high-risk group than in the low-risk group, but this difference was not statistically significant (Fig. 4A). The immune score was significantly lower in the high-risk group than in the low-risk group (Fig. 4B). There was no significant difference in the estimated score between the high-risk and low-risk groups (Fig. 4C), indicating that the two groups' tumor compositions were similar. Among the eight glycolysis-related prognostic genes, the expression levels of *DCN* and *FBP1* positively correlated with the immune score (Fig. 4D, Additional file 2: Table S2).

### GSEA of low-risk and high-risk groups

**Fig. 5:**
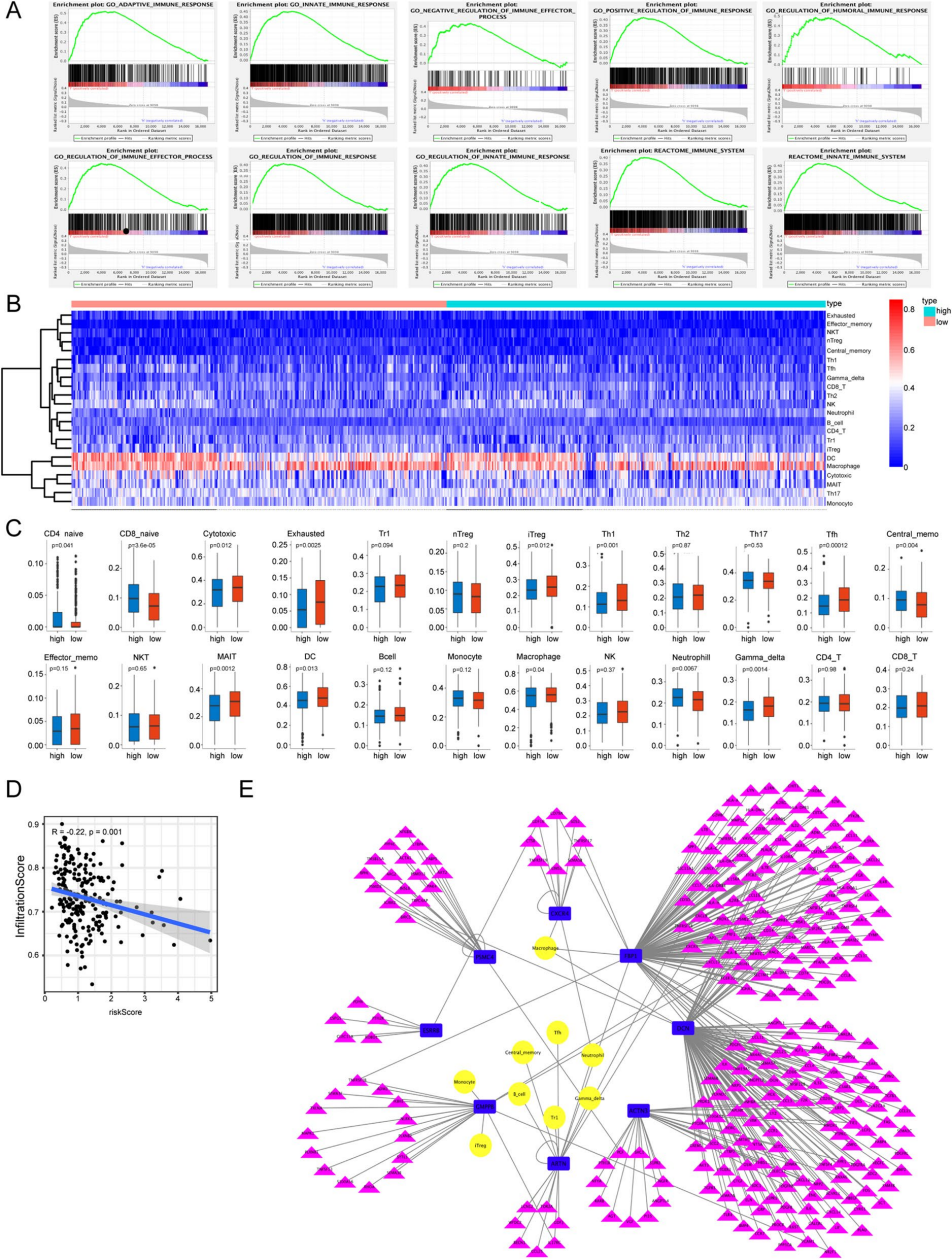
Immune analysis of between the high-risk group and the low-risk group. **A** Multiple immune-related gene sets significantly enriched in the low-risk group determined by GSEA. The immune-related gene sets included: O_ADAPTIVE_IMMUNE_RESPONSE, GO_INNATE_IMMUNE_RESPONSE, GO_NEGATIVE_REGULATION_OF_IMMUNE_EFFECTOR_PROCESS, GO_POSITIVE_REGULATION_OF_IMMUNE_RESPONSE, GO_REGULATION_OF_HUMORAL_IMMUNE_RESPONSE, GO_REGULATION_OF_IMMUNE_EFFECTOR_PROCESS, GO_REGULATION_OF_IMMUNE_RESPONSE, GO_REGULATION_OF_INNATE_IMMUNE_RESPONSE, and REACTOME_IMMUNE_SYSTEM, REACTOME_INNATE_IMMUNE_SYSTEM. **B** Heatmap of the immune cell composition in the entire cohort. **C** Comparison of the immune cell composition between the high-risk and low-risk groups. **D** Correlation analysis of immune infiltration scores and risk scores (*p* < 0.05). **E** Network of immune-related genes, immune cells, and eight glycolysis-related prognostic genes. (Blue rectangles: glycolysis-related prognostic genes, yellow circles: immune cells, purple triangles: immune-related genes)

GSEA was performed to investigate the relationship between the risk level in ovarian cancer and an array of immune functions. As expected, the low-risk group was significantly enriched in multiple immune-related gene sets (*p* < 0.05) (Fig. 5A).

### Relationship between the glycolytic risk model and immune cell infiltration

Our results indicated that immune cell composition and immune-related genes in the tumors differed between the high-risk and low-risk groups. Accordingly, we evaluated the relationship between glycolysis and immune cell infiltration. We uploaded the entire cohort’s transcriptome data to ImmuCellAI to analyze each group’s immune cell composition (Fig. 5B). In total, 13 of 24 immune cell types differed between the groups, including naive CD4 T cells, naive CD8 T cells, cytotoxic T cells, exhausted T cells, inducible regulatory T cells (iTreg), helper T cells 1 (Th1), follicular helper T cells (Tfh), central memory T cells (Tcm), mucosal‐associated invariant T cells (MAIT), DCs, Macrophages, Neutrophils, and gamma delta T cells (γδ T) (Fig. 5C). Correlations between the expression levels of the eight risk genes and immune cell components were also evaluated (Additional file 2: Table S3). The expression of *ARTN* was related to cellular components, including neutral and central_ memory, Gamma_ delta, Tr1, B_ Cell, and Tfh. Moreover, the risk score negatively correlated with the immune infiltration score (R = − 0.22) (Fig. 5D).

To further investigate the roles of the eight prognostic genes in immune infiltration, the immune gene set was extracted from ImmPort. Among these eight genes, *ARTN*, *PSMC4*, and *CXCR4* were identified to be involved in glycolysis and immune function. The correlations between immune-related gene expression and eight glycolysis-related risk genes in ovarian cancer were analyzed (Additional file 2: Table S4). Many immune-related genes were co-expressed with *DCN* (130 genes), followed by *FBP1* (114 genes). Further, a network diagram of immune cells, immune-related genes, and the eight prognostic genes has been generated (Fig. 5E).

## Discussion

Metabolic reprogramming is essential for tumor cell growth, survival, and proliferation. Recent research has focused on glycolytic processes in cell proliferation, invasion, autophagy, and chemotherapy resistance in ovarian cancer. For example, Zhao et al. [10] have shown that mitochondrial elongation factor 2 (*MIEF2*) overexpression promotes growth and metastasis in ovarian cancer by reprogramming glucose metabolism. The knockdown of *DIO3* in high-grade serous ovarian cancer leads to a decrease in the Warburg effect and carcinogenic signal transduction and tumor growth inhibition [11]. By reversing the Warburg effect, ABT737, a B cell lymphoma (BCL2) inhibitor, promotes H_2_O_2_-induced apoptosis and increases the antitumor effect of oxidative stress, sensitizing ovarian cancer cells to chemotherapy [12]. Aurora-A, a member of the Aurora kinase family, regulates glucose metabolism in ovarian cancer via the SOX8/FOXK1 signaling axis and induces cisplatin resistance [13]. The inhibition of Anexelekto (AXL), a member of the tyro3-axl-mer family of receptor tyrosine kinases, impairs glycolysis in cisplatin-resistant ovarian cancer cells [14].

Despite extensive research on prognostic signatures in ovarian cancer, studies involving glycolysis-related genes with a predictive significance are lacking. To better predict survival rates, we analyzed TCGA and GEO datasets to establish a glycolysis-related risk model. Using multiple datasets with random assignment to the training and test sets minimizes biases associated with individual data sources. Using univariate and multivariate Cox analyses, we confirmed that the newly developed model was an independent prognostic indicator for ovarian cancer. The prediction ability of the model exceeded that of other clinical features, as evaluated by ROC curves. The results were verified using the test set. Risk scores for all cases were calculated and analyzed concerning the tumor microenvironment and immunity in both groups.

Various glycolysis-related prognostic genes in ovarian cancer, including *ACTN3*, *ARTN*, *CXCR4*, *DCN*, *ESRRB*, *FBP1*, *GMPPB*, and *PSMC4*, were identified in this study. Most of these genes have been studied in a variety of tumors. *ACTN3* encodes actinin 3, a cytoskeletal molecule of actin filament cross-linked protein 5, mainly occurring in the skeletal muscle. In tumors, it interacts with parafibromin tumor suppressor protein, which is involved in the hypermethylation and inhibition of many oncogenes [15].

As a member of the glial cell line-derived neurotrophic factor ligand family, *ARTN* is associated with many malignant tumors [16, 17]. In liver cancer, artemin-positive, tumor-inducible, erythroblast-like cells (Ter-cells) could promote tumor progression, whereas an artemin deficiency abolishes the tumor-promoting effect [18]. Besides, *ARTN* is associated with chemoresistance in a variety of tumors. For example, in endometrial cancer, *ARTN* specifically regulates CD24 to stimulate endometrial cancer cell resistance to doxorubicin and paclitaxel [19].

*CXCR4* is a member of the CXC chemokine receptor family, involved in regulating cancer progression by binding to unique ligands in the tumor microenvironment. The anti-*CXCR4* single-chain variable fragment antibody isolated by Liang et al. [20] inhibits proliferation and angiogenesis and increases apoptosis in tumor cells. Compared to healthy renal cells, the expression of *CXCR4* is significantly increased in clear cell renal cell carcinoma, and this increased expression is significantly correlated with tumor stage or grade, thus indicating significant predictive value [21].

*DCN* is a leucine-rich proteoglycan in the extracellular matrix. It inhibits tumor growth and migration in hepatocellular carcinoma [22], colon cancer [23], and prostate cancer [24]. However, in gastric cancer [25], head and neck cancer [26], bladder cancer [27], and endometrial cancer [28], *DCN* is a predictor of poor prognosis, as determined by bioinformatics analyses.

*ESRRB*, a stem cell marker, plays a crucial role in regulating the immature pluripotent state. It is highly expressed in colon cancer, prostate cancer, and bladder cancer [29]. Furthermore, in glioblastoma, its activation limits tumor migration and intracranial tumor growth [30]. However, similar to *DCN*, *ESRRB* has antitumor effects in some tumors. For example, it is a negative regulator of the cell cycle in breast cancer, and its expression is negatively related to the expression of transcription inhibitor enhancer of zeste 2 polycomb repressive complex 2 subunits [31].

*FBP1* is an enzyme involved in the catalytic conversion of fructose-1,6-diphosphate to fructose-6-phosphate. The abnormal expression or loss of function of *FBP1* has been observed in breast [32] and esophageal cancer [33]. *FBP1* deletion leads to steatosis, accompanied by the activation and senescence of hepatic stellate cells, which show senescence-related secretory phenotypes [34]. Low *FBP1* expression has been found in patients with postoperative recurrence of prostate cancer [35].

*GMPPB* is an important enzyme affecting the O-glycosylation of alpha-dystroglycan and N-glycosylation of beta-dystroglycan, and little is known about its role in tumor biology. Only two related studies indicate that *GMPPB* could predict prognosis in endometrial cancer [28].

The glycolytic gene *PSMC4* is also an immune-related gene. In previous bioinformatics studies, *PSMC4* has been identified as part of the immune gene signature for predicting prognosis in endometrial cancer [36]. Moreover, Ayakannu et al. [37] confirmed that *PSMC4* is one of the most “stable” endogenous control genes in type I endometrial cancer using geNorm Qbase + 2 and NormFinder software package. However, none of the eight prognostic genes have been studied in ovarian cancer.

The lactic acid produced by glycolysis accumulates outside the cells, which reduces tissue pH and affects the tumor microenvironment. The acidic tumor microenvironment confers the tumor with resistance to treatment and remains the main obstacle to the successful treatment of cancer [38]. In this study, the high-risk group's immune score was significantly lower than that of the low-risk group, suggesting a greater potential for immune escape in the high-risk group than in the low-risk group. Furthermore, through concentration gradient-dependent inhibition, excessive lactic acid in the tumor microenvironment inhibits T cells from releasing lactic acid, thereby affecting T cells' function and proliferation [39]. Our results revealed that 13 types of immune cells differed between the low-risk and high-risk groups, among which 10 were T cells, including CD4_naive, iTreg, Th1, Tfh, CD8_naive, exhausted, MAIT, cytotoxic, central_meno, and Gamma_delta. In addition to T cells, NK cells and myeloid-derived suppressor cells (MDSCs) are affected by lactic acid, resulting in significant activity changes [40]. MDSCs are precursors of DCs, macrophages, and granulocytes. In ovarian cancer, we found that the composition of DCs, macrophages, and neutrophils differed significantly between high-risk and low-risk groups. These findings suggest that glycolysis affects the composition of immune cell populations in ovarian cancer.

Although considerable research supports the relationship between tumor glycolysis and immune escape from the tumor microenvironment, the specific molecules contributing to this relationship have not been determined. This study found that the expression levels of the glycolytic genes *DCN* and *FBP1* positively correlated with the ovarian tumor microenvironment's immune score. Several immune-related genes are associated with the expression of these two glycolytic genes involved in macrophages infiltration. Also, the glycolytic genes *ARTN*, *PSMC4*, and *CXCR4* are immune-related. In particular, *ARTN* correlates with the infiltration of six immune cell types. Although there is limited research on how these molecules regulate immune processes in ovarian cancer, our findings revealed the molecules involved in both glycolysis and immune processes in ovarian cancer might represent potential targets for developing novel treatment strategies. In particular, future studies should evaluate the effectiveness of a “two-hit” method integrating glycolysis and immune escape or infiltration characteristics.

To our knowledge, our study is the first to establish a model for predicting the prognosis of ovarian cancer, depending on the expression of glycolysis-related genes. In clinical settings, one can easily measure the expression of these eight prognostic signature genes in ovarian cancer samples to calculate patients’ risk score and predict their survival rate. The prognostic model and nomogram established herein are not only easy to use, economical, and practical, but also have good prospects for clinical application. It is also the first to explore the relationship between glycolysis-related genes and immune genes in the ovarian cancer microenvironment. However, this study was limited by the lack of healthy tissue samples in the TCGA and GEO datasets and the lack of data on the precise mechanisms by which these eight glycolytic genes regulate glycolysis and immunity in ovarian cancer. In our future research, we plan to focus on these mechanisms.

## Conclusions

In conclusion, we identified eight glycolysis-related prognostic genes in ovarian cancer, establishing a predictive model for the practical estimation of survival rates. Our results also revealed a correlation between glycolysis and immune function in ovarian cancer, indicating that the eight prognostic genes may also contribute to immune processes. These findings provide potential biomarkers and therapeutic targets for ovarian cancer.

## Supplementary Information

**Additional file 1:Fig S1.** A-B: Lasso regression analysis of glycolysis-related genes with prognostic value in ovarian cancer based on the training set.

**Additional file 2:Table S1.** Clinical features of 581 patients with ovarian cancer in TCGA database and the GEO datasets. **Table S2.** Correlation analysis of immune scores and eight glycolysis-related prognostic genes. **Table S3.** Correlation analysis of immune cell components and eight glycolysis-related prognostic genes. **Table S4.** Correlation analysis of immune-related genes and eight glycolysis-related prognostic genes.

## Data Availability

The datasets generated and/or analysed during the current study are available in the TCGA (https://portal.gdc.cancer.gov/) and GEO (https://www.ncbi.nlm.nih.gov/geo/) (GSE17260; GSE73614). The data that support the findings of this study are available from the corresponding author upon reasonable request.

## Abbreviations

GSEA: Gene set enrichment analysis
TCGA: The Cancer Genome Atlas
GEO: Gene Expression Omnibus
SLC2A1: Solute carrier family 2 member 1
MSigDB: Molecular Signatures Database
ACTN3: Actinin alpha 3
ARTN: Artemin
CXCR4: CXC motif chemokine receptor 4
DCN: Decorin
ESRRB: Estrogen-related receptor beta
FBP1: Fructose-bisphosphatase 1
GMPPB: GDP-mannose pyrophosphorylase B
PSMC4: Proteasome 26S subunit, ATPase 4
iTreg: Inducible regulatory T cells
Th1: Helper T cells
Tfh: Follicular helper T cells
Tcm: Central memory T cells
MAIT: Mucosal‐associated invariant T cells
γδ T: Gamma delta T cells
MIEF2: Mitochondrial elongation factor 2
BCL2: B cell lymphoma
AXL: Anexelekto
Ter-cells: Erythroblast-like cells
MDSCs: Myeloid-derived suppressor cells
