# Supplementary figures and images for: Establishment of a novel glycolysis-related prognostic gene signature for ovarian cancer and its relationships with immune infiltration of the tumor microenvironment
Source: J Transl Med. 2021 Sep 8;19:382. doi: 10.1186/s12967-021-03057-0 (PMC8425093; doi:10.1186/s12967-021-03057-0)

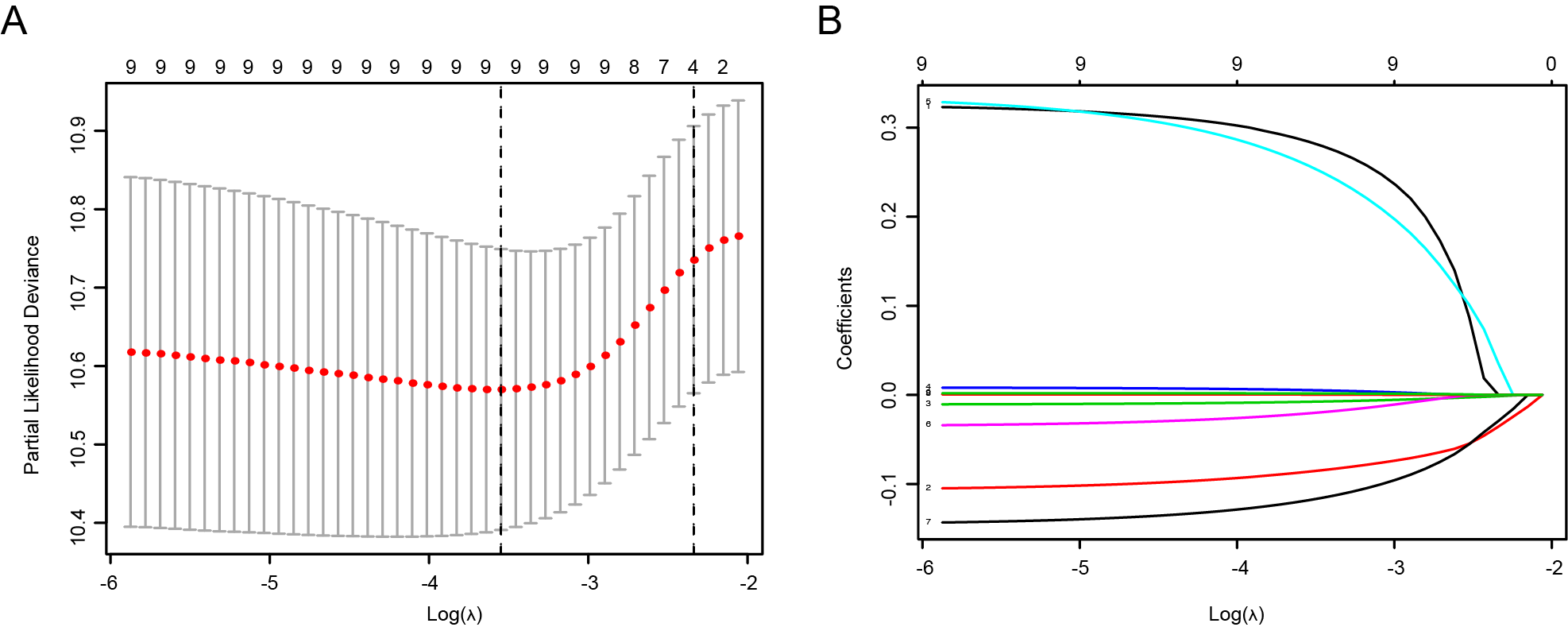

Supplement: Supplementary file 1 — Additional file 1:Fig S1. A-B: Lasso regression analysis of glycolysis-related genes with prognostic value in ovarian cancer based on the training set. [file 12967_2021_3057_MOESM1_ESM.png]
