# Supplementary material for: Establishment of a novel glycolysis-related prognostic gene signature for ovarian cancer and its relationships with immune infiltration of the tumor microenvironment
Source: J Transl Med. 2021 Sep 8;19:382. doi: 10.1186/s12967-021-03057-0 (PMC8425093; doi:10.1186/s12967-021-03057-0)
Supplement: Supplementary file 2 — Additional file 2:Table S1. Clinical features of 581 patients with ovarian cancer in TCGA database and the GEO datasets. Table S2. Correlation analysis of immune scores and eight glycolysis-related prognostic genes. Table S3. Correlation analysis of immune cell components and eight glycolysis-related prognostic genes. Table S4. Correlation analysis of immune-related genes and eight glycolysis-related prognostic genes. [file 12967_2021_3057_MOESM2_ESM.docx]

Table S1: Clinical features of 581 patients with ovarian cancer in TCGA database and the GEO datasets.

| **Clinical character** | | | **TCGA** | | | **GSE17260** | | | **GSE73614** | | | **Training**  **set** | **Test**  **set** |
| --- | --- | --- | --- | --- | --- | --- | --- | --- | --- | --- | --- | --- | --- |
|  |  |  | **Training**  **set** | **Test**  **set** | **All**  **set** | **Training**  **set** | **Test**  **set** | **All**  **set** | **Training**  **set** | **Test**  **set** | **All**  **set** |  |  |
| Grade | G1 | | 1 | 0 | 1 | 15 | 11 | 26 | 0 | 0 | 0 | 16 | 11 |
|  | G2 | | 23 | 19 | 42 | 19 | 22 | 41 | 13 | 16 | 29 | 55 | 57 |
|  | G3 | | 154 | 157 | 311 | 21 | 22 | 43 | 36 | 33 | 69 | 211 | 212 |
|  | unknow | | 5 | 5 | 10 | 0 | 0 | 0 | 5 | 4 | 9 | 10 | 9 |
| Stage | I | a | 0 | 0 | 0 | 0 | 0 | 0 | 7 | 5 | 12 | 7 | 5 |
|  |  | b | 0 | 0 | 0 | 0 | 0 | 0 | 1 | 0 | 1 | 1 | 0 |
|  |  | c | 1 | 0 | 1 | 0 | 0 | 0 | 8 | 11 | 19 | 9 | 11 |
|  | II | a | 3 | 0 | 3 | 0 | 0 | 0 | 1 | 0 | 1 | 4 | 0 |
|  |  | b | 3 | 0 | 3 | 0 | 0 | 0 | 0 | 1 | 1 | 3 | 1 |
|  |  | c | 7 | 8 | 15 | 0 | 0 | 0 | 5 | 9 | 14 | 12 | 17 |
|  | III | a | 5 | 2 | 7 | 3 | 3 | 6 | 0 | 3 | 3 | 8 | 8 |
|  |  | b | 5 | 8 | 13 | 12 | 6 | 18 | 2 | 3 | 5 | 19 | 17 |
|  |  | c | 126 | 139 | 265 | 33 | 36 | 69 | 26 | 15 | 41 | 185 | 190 |
|  | IV |  | 31 | 23 | 54 | 7 | 10 | 17 | 4 | 6 | 10 | 42 | 39 |
|  | unknow | | 2 | 1 | 3 | 0 | 0 | 0 | 0 | 0 | 0 | 2 | 1 |
| Vital status | Alive | | 90 | 78 | 168 | 36 | 28 | 64 | 24 | 25 | 49 | 150 | 131 |
|  | Dead | | 93 | 103 | 196 | 19 | 27 | 46 | 30 | 28 | 58 | 142 | 158 |
| Survival time (day) | ≤365 | | 12 | 10 | 22 | 4 | 5 | 9 | 7 | 2 | 9 | 23 | 17 |
|  | 366-730 | | 23 | 19 | 42 | 2 | 5 | 7 | 7 | 7 | 14 | 32 | 31 |
|  | 731-1095 | | 16 | 21 | 37 | 4 | 10 | 14 | 3 | 5 | 8 | 23 | 36 |
|  | 1096-1460 | | 21 | 23 | 44 | 3 | 1 | 4 | 1 | 4 | 5 | 25 | 28 |
|  | 1461-1825 | | 10 | 15 | 25 | 2 | 5 | 7 | 3 | 2 | 5 | 15 | 22 |
|  | ≥1826 | | 11 | 15 | 26 | 4 | 1 | 5 | 9 | 8 | 17 | 24 | 24 |
| follow-up time（day） | ≤365 | | 29 | 36 | 65 | 1 | 0 | 1 | 0 | 2 | 2 | 30 | 38 |
|  | 366-730 | | 12 | 10 | 22 | 11 | 7 | 18 | 1 | 1 | 2 | 24 | 18 |
|  | 731-1095 | | 21 | 8 | 29 | 7 | 7 | 14 | 1 | 3 | 4 | 29 | 18 |
|  | 1096-1460 | | 11 | 6 | 17 | 6 | 4 | 10 | 0 | 1 | 1 | 17 | 11 |
|  | 1461-1825 | | 6 | 5 | 11 | 4 | 4 | 8 | 1 | 3 | 4 | 11 | 12 |
|  | ≥1826 | | 11 | 13 | 24 | 7 | 6 | 13 | 21 | 15 | 36 | 39 | 34 |
| **Total number** | | |  |  | **364** |  |  | **110** |  |  | **107** | **292** | **289** |

Table S2. Correlation analysis of immune scores and eight glycolysis-related prognostic genes.

| gene | corr | corPval |
| --- | --- | --- |
| FBP1 | 0.315259334 | 7.14E-15 |
| DCN | 0.306712896 | 4.03E-14 |
| ESRRB | -0.121742158 | 0.003291824 |
| GMPPB | 0.121729209 | 0.00329517 |
| CXCR4 | 0.107369973 | 0.009598799 |
| ARTN | 0.077080462 | 0.063353172 |
| PSMC4 | -0.064656731 | 0.119528675 |
| ACTN3 | 0.04174395 | 0.315154689 |

Table S3. Correlation analysis of immune cell components and eight glycolysis-related prognostic genes.

| gene | immune cell | corr | corPval |
| --- | --- | --- | --- |
| ARTN | Tfh | 0.38247833 | 5.72E-09 |
| ARTN | B_cell | 0.27604803 | 3.73E-05 |
| ARTN | Tr1 | 0.23941269 | 0.00037328 |
| ARTN | Neutrophil | -0.2138489 | 0.00153105 |
| ARTN | Central_memory | -0.2074394 | 0.00212911 |
| ARTN | Gamma_delta | 0.2010231 | 0.00293393 |
| CXCR4 | Neutrophil | -0.2771047 | 3.48E-05 |
| CXCR4 | Macrophage | 0.265314 | 7.59E-05 |
| CXCR4 | InfiltrationScore | 0.24667667 | 0.00024288 |
| DCN | Macrophage | 0.24617361 | 0.00025032 |
| FBP1 | Macrophage | 0.20928765 | 0.00193787 |
| GMPPB | iTreg | 0.21948507 | 0.00113665 |
| GMPPB | Monocyte | 0.21605648 | 0.00136366 |

Table S4. Correlation analysis of immune-related genes and eight glycolysis-related prognostic genes.

| glycolytic gene | immune gene | corr | p-value | Regulation |
| --- | --- | --- | --- | --- |
| ACTN3 | AGT | 0.21620698 | 1.42E-07 | positive |
| ACTN3 | RARB | 0.21826954 | 1.07E-07 | positive |
| ACTN3 | PDGFRB | 0.22906993 | 2.35E-08 | positive |
| ACTN3 | NFYA | 0.23129825 | 1.70E-08 | positive |
| ACTN3 | PTH1R | 0.23317581 | 1.29E-08 | positive |
| ACTN3 | ACVRL1 | 0.24597964 | 1.87E-09 | positive |
| ACTN3 | PDGFRA | 0.25928645 | 2.22E-10 | positive |
| ACTN3 | HMOX1 | 0.26402017 | 1.01E-10 | positive |
| ACTN3 | COLEC12 | 0.26655677 | 6.59E-11 | positive |
| ACTN3 | LRP1 | 0.26786639 | 5.27E-11 | positive |
| ACTN3 | ANXA6 | 0.27670124 | 1.14E-11 | positive |
| ACTN3 | CXCL12 | 0.2957603 | 3.41E-13 | positive |
| ACTN3 | SLIT2 | 0.30337578 | 7.80E-14 | positive |
| ACTN3 | SHC1 | 0.30875432 | 2.68E-14 | positive |
| ACTN3 | TIE1 | 0.30911239 | 2.49E-14 | positive |
| ACTN3 | PGF | 0.31298086 | 1.14E-14 | positive |
| ACTN3 | S1PR2 | 0.31516589 | 7.28E-15 | positive |
| ACTN3 | NRP2 | 0.32109598 | 2.12E-15 | positive |
| ACTN3 | NGFR | 0.3905679 | 1.30E-22 | positive |
| ACTN3 | DES | 0.40138008 | 6.70E-24 | positive |
| ACTN3 | CD209 | 0.42633865 | 4.63E-27 | positive |
| ACTN3 | ANGPTL4 | 0.44535599 | 1.18E-29 | positive |
| ACTN3 | EDNRA | 0.4545547 | 5.75E-31 | positive |
| ACTN3 | PI15 | 0.4606401 | 7.39E-32 | positive |
| ACTN3 | VGF | 0.6488597 | 9.97E-71 | positive |
| ARTN | BECN1 | -0.2042458 | 6.87E-07 | negative |
| ARTN | TNFRSF18 | 0.20453761 | 6.62E-07 | positive |
| ARTN | WFDC2 | 0.21070214 | 2.97E-07 | positive |
| ARTN | LCN12 | 0.21709438 | 1.26E-07 | positive |
| ARTN | TOR2A | 0.23020729 | 1.99E-08 | positive |
| ARTN | GDF3 | 0.2340551 | 1.14E-08 | positive |
| ARTN | CXCR3 | 0.2491258 | 1.14E-09 | positive |
| ARTN | IL17RC | 0.2562893 | 3.63E-10 | positive |
| ARTN | CCL25 | 0.29272429 | 6.07E-13 | positive |
| ARTN | ARTN | 0.93637868 | 1.89E-265 | positive |
| CXCR4 | TNFRSF19 | 0.20098434 | 1.04E-06 | positive |
| CXCR4 | CTSB | 0.20348458 | 7.57E-07 | positive |
| CXCR4 | GDF10 | 0.23321652 | 1.29E-08 | positive |
| CXCR4 | CD79A | 0.25338064 | 5.80E-10 | positive |
| CXCR4 | SOS1 | 0.25856442 | 2.50E-10 | positive |
| CXCR4 | TNFRSF17 | 0.25907774 | 2.30E-10 | positive |
| CXCR4 | SEMA5B | 0.29958157 | 1.64E-13 | positive |
| CXCR4 | LIMS1 | 0.35459031 | 1.18E-18 | positive |
| CXCR4 | CXCR4 | 0.91466066 | 6.20E-230 | positive |
| DCN | RORC | -0.2598122 | 2.04E-10 | negative |
| DCN | FAM3B | -0.2010088 | 1.04E-06 | negative |
| DCN | INPP5D | 0.20008852 | 1.16E-06 | positive |
| DCN | CCR7 | 0.205592 | 5.78E-07 | positive |
| DCN | ARRB1 | 0.20625959 | 5.30E-07 | positive |
| DCN | TLR2 | 0.20697449 | 4.83E-07 | positive |
| DCN | CCL19 | 0.20963678 | 3.42E-07 | positive |
| DCN | LIF | 0.21178342 | 2.57E-07 | positive |
| DCN | FOS | 0.21321375 | 2.13E-07 | positive |
| DCN | DES | 0.21410417 | 1.89E-07 | positive |
| DCN | ANGPTL1 | 0.21443733 | 1.80E-07 | positive |
| DCN | NRP2 | 0.21467696 | 1.75E-07 | positive |
| DCN | CCR1 | 0.2152599 | 1.61E-07 | positive |
| DCN | HBEGF | 0.21626927 | 1.41E-07 | positive |
| DCN | LYZ | 0.21826149 | 1.07E-07 | positive |
| DCN | TPM2 | 0.21830832 | 1.07E-07 | positive |
| DCN | CD14 | 0.21959031 | 8.94E-08 | positive |
| DCN | CD86 | 0.21986599 | 8.61E-08 | positive |
| DCN | WNT5A | 0.22005069 | 8.39E-08 | positive |
| DCN | PPP3CA | 0.22132679 | 7.03E-08 | positive |
| DCN | CCR5 | 0.22195831 | 6.44E-08 | positive |
| DCN | CD48 | 0.22321874 | 5.40E-08 | positive |
| DCN | PTGER3 | 0.22517346 | 4.10E-08 | positive |
| DCN | IL33 | 0.22524626 | 4.06E-08 | positive |
| DCN | ICAM2 | 0.22617573 | 3.56E-08 | positive |
| DCN | SLIT2 | 0.22763135 | 2.89E-08 | positive |
| DCN | CCL18 | 0.2279893 | 2.75E-08 | positive |
| DCN | CYBB | 0.22916265 | 2.32E-08 | positive |
| DCN | CSF2RA | 0.23033558 | 1.96E-08 | positive |
| DCN | GRP | 0.23392006 | 1.16E-08 | positive |
| DCN | NR4A3 | 0.2345206 | 1.06E-08 | positive |
| DCN | IL6ST | 0.2346532 | 1.04E-08 | positive |
| DCN | PTGS2 | 0.23844725 | 5.91E-09 | positive |
| DCN | FAM19A5 | 0.23910752 | 5.35E-09 | positive |
| DCN | SEMA5A | 0.23941924 | 5.10E-09 | positive |
| DCN | TNFSF13B | 0.24057609 | 4.28E-09 | positive |
| DCN | FCGR2B | 0.24111348 | 3.95E-09 | positive |
| DCN | LCP2 | 0.2414803 | 3.73E-09 | positive |
| DCN | CD209 | 0.24167359 | 3.63E-09 | positive |
| DCN | PTGER4 | 0.24173796 | 3.59E-09 | positive |
| DCN | FGR | 0.24245524 | 3.22E-09 | positive |
| DCN | S100A12 | 0.24378168 | 2.62E-09 | positive |
| DCN | ILK | 0.24381175 | 2.61E-09 | positive |
| DCN | NR4A1 | 0.24738567 | 1.50E-09 | positive |
| DCN | OSM | 0.2496396 | 1.05E-09 | positive |
| DCN | PLXND1 | 0.25076268 | 8.81E-10 | positive |
| DCN | CSF1R | 0.25165675 | 7.64E-10 | positive |
| DCN | CCL4 | 0.25394086 | 5.30E-10 | positive |
| DCN | VDR | 0.25606282 | 3.76E-10 | positive |
| DCN | ITGB2 | 0.25667593 | 3.41E-10 | positive |
| DCN | CCL3 | 0.25895707 | 2.35E-10 | positive |
| DCN | ELN | 0.25944029 | 2.17E-10 | positive |
| DCN | PPARG | 0.26487158 | 8.76E-11 | positive |
| DCN | BMP2 | 0.26583633 | 7.44E-11 | positive |
| DCN | FCGR3A | 0.26614503 | 7.07E-11 | positive |
| DCN | CSF2RB | 0.2714237 | 2.86E-11 | positive |
| DCN | HCK | 0.2720397 | 2.57E-11 | positive |
| DCN | IL10RA | 0.27263943 | 2.32E-11 | positive |
| DCN | CALCRL | 0.27295341 | 2.19E-11 | positive |
| DCN | PLA2G2A | 0.27549988 | 1.40E-11 | positive |
| DCN | NR2F1 | 0.27910421 | 7.41E-12 | positive |
| DCN | SDC2 | 0.27924353 | 7.22E-12 | positive |
| DCN | ANXA6 | 0.28299332 | 3.68E-12 | positive |
| DCN | VEGFC | 0.28470044 | 2.69E-12 | positive |
| DCN | IGF1 | 0.28693143 | 1.79E-12 | positive |
| DCN | SDC1 | 0.28933564 | 1.15E-12 | positive |
| DCN | IL6 | 0.28982375 | 1.05E-12 | positive |
| DCN | MSR1 | 0.29265309 | 6.15E-13 | positive |
| DCN | OLR1 | 0.29290463 | 5.87E-13 | positive |
| DCN | BMP4 | 0.29617367 | 3.15E-13 | positive |
| DCN | PDGFC | 0.30153811 | 1.12E-13 | positive |
| DCN | PDGFB | 0.30276413 | 8.80E-14 | positive |
| DCN | C3AR1 | 0.3045265 | 6.22E-14 | positive |
| DCN | HMOX1 | 0.30679375 | 3.97E-14 | positive |
| DCN | TNFSF4 | 0.31125639 | 1.62E-14 | positive |
| DCN | SEMA6B | 0.31135965 | 1.58E-14 | positive |
| DCN | NRP1 | 0.31272991 | 1.20E-14 | positive |
| DCN | PTPRC | 0.31478321 | 7.88E-15 | positive |
| DCN | CMKLR1 | 0.316696 | 5.31E-15 | positive |
| DCN | GMFG | 0.31772097 | 4.29E-15 | positive |
| DCN | FPR1 | 0.32244367 | 1.60E-15 | positive |
| DCN | TGFB1 | 0.32310742 | 1.39E-15 | positive |
| DCN | TIE1 | 0.325281 | 8.74E-16 | positive |
| DCN | FABP4 | 0.32841317 | 4.46E-16 | positive |
| DCN | CCL21 | 0.34660661 | 7.65E-18 | positive |
| DCN | BMP1 | 0.35186206 | 2.25E-18 | positive |
| DCN | AKT3 | 0.36231183 | 1.84E-19 | positive |
| DCN | PROK1 | 0.36424832 | 1.14E-19 | positive |
| DCN | ENG | 0.36497936 | 9.54E-20 | positive |
| DCN | FAS | 0.36658713 | 6.41E-20 | positive |
| DCN | PROCR | 0.37058018 | 2.37E-20 | positive |
| DCN | PLAUR | 0.38200302 | 1.26E-21 | positive |
| DCN | SEMA7A | 0.38684275 | 3.53E-22 | positive |
| DCN | TNFSF12 | 0.39520153 | 3.70E-23 | positive |
| DCN | C5AR1 | 0.40097035 | 7.52E-24 | positive |
| DCN | TGFB3 | 0.4019394 | 5.73E-24 | positive |
| DCN | CTGF | 0.40662479 | 1.53E-24 | positive |
| DCN | VIM | 0.41223359 | 3.05E-25 | positive |
| DCN | SEMA3C | 0.42029571 | 2.85E-26 | positive |
| DCN | LTBP2 | 0.4233566 | 1.14E-26 | positive |
| DCN | LRP1 | 0.42613176 | 4.93E-27 | positive |
| DCN | F2R | 0.44044568 | 5.74E-29 | positive |
| DCN | TGFBR2 | 0.4411142 | 4.63E-29 | positive |
| DCN | TLR4 | 0.44467527 | 1.48E-29 | positive |
| DCN | S1PR1 | 0.44944672 | 3.12E-30 | positive |
| DCN | ACVRL1 | 0.45269039 | 1.07E-30 | positive |
| DCN | IL7R | 0.46606712 | 1.14E-32 | positive |
| DCN | CXCL14 | 0.46815805 | 5.52E-33 | positive |
| DCN | PLXNC1 | 0.46867904 | 4.60E-33 | positive |
| DCN | CYR61 | 0.46900729 | 4.10E-33 | positive |
| DCN | APLNR | 0.47751031 | 2.00E-34 | positive |
| DCN | CMTM3 | 0.4878751 | 4.49E-36 | positive |
| DCN | GREM1 | 0.50177157 | 2.24E-38 | positive |
| DCN | PDGFRL | 0.5058345 | 4.54E-39 | positive |
| DCN | CCL11 | 0.5134779 | 2.12E-40 | positive |
| DCN | OGN | 0.51521109 | 1.05E-40 | positive |
| DCN | APOD | 0.51536252 | 9.86E-41 | positive |
| DCN | PLAU | 0.51958832 | 1.74E-41 | positive |
| DCN | SCT | 0.52096367 | 9.81E-42 | positive |
| DCN | PDGFD | 0.53314512 | 5.57E-44 | positive |
| DCN | SCG2 | 0.55111927 | 1.82E-47 | positive |
| DCN | THBS1 | 0.55332201 | 6.60E-48 | positive |
| DCN | VCAM1 | 0.58107828 | 9.10E-54 | positive |
| DCN | EDNRA | 0.60172349 | 1.66E-58 | positive |
| DCN | INHBA | 0.61338688 | 2.45E-61 | positive |
| DCN | COLEC12 | 0.62916728 | 2.30E-65 | positive |
| DCN | ANGPTL2 | 0.63453581 | 8.67E-67 | positive |
| DCN | CXCL12 | 0.64778198 | 2.01E-70 | positive |
| DCN | PDGFRB | 0.64827611 | 1.46E-70 | positive |
| DCN | PDGFRA | 0.70240246 | 1.52E-87 | positive |
| ESRRB | CSPG5 | 0.21391078 | 1.94E-07 | positive |
| ESRRB | ROBO1 | 0.21653251 | 1.36E-07 | positive |
| ESRRB | PTH2R | 0.2187105 | 1.01E-07 | positive |
| ESRRB | GDF10 | 0.22087509 | 7.49E-08 | positive |
| ESRRB | PDYN | 0.26608092 | 7.14E-11 | positive |
| ESRRB | CLEC11A | 0.26893502 | 4.39E-11 | positive |
| FBP1 | CXCL10 | 0.20087833 | 1.05E-06 | positive |
| FBP1 | SECTM1 | 0.2043187 | 6.81E-07 | positive |
| FBP1 | IL2RB | 0.20484206 | 6.37E-07 | positive |
| FBP1 | NFKBIE | 0.20733502 | 4.61E-07 | positive |
| FBP1 | TNFRSF4 | 0.20836795 | 4.03E-07 | positive |
| FBP1 | ARTN | 0.20912626 | 3.65E-07 | positive |
| FBP1 | HLA-H | 0.20919706 | 3.62E-07 | positive |
| FBP1 | PTK2B | 0.21094308 | 2.88E-07 | positive |
| FBP1 | IL4R | 0.21112952 | 2.80E-07 | positive |
| FBP1 | CSF1R | 0.21172958 | 2.59E-07 | positive |
| FBP1 | CCL3 | 0.21287865 | 2.22E-07 | positive |
| FBP1 | TNFSF13B | 0.21425573 | 1.85E-07 | positive |
| FBP1 | IL7R | 0.21573271 | 1.51E-07 | positive |
| FBP1 | LTB | 0.21722054 | 1.24E-07 | positive |
| FBP1 | IL3RA | 0.21853169 | 1.03E-07 | positive |
| FBP1 | IL1RN | 0.21941897 | 9.16E-08 | positive |
| FBP1 | MSR1 | 0.22131402 | 7.04E-08 | positive |
| FBP1 | PSMB8 | 0.22140883 | 6.95E-08 | positive |
| FBP1 | TNFRSF14 | 0.22362286 | 5.10E-08 | positive |
| FBP1 | PSME2 | 0.22705692 | 3.14E-08 | positive |
| FBP1 | LYN | 0.22715305 | 3.09E-08 | positive |
| FBP1 | HLA-E | 0.23069908 | 1.86E-08 | positive |
| FBP1 | TNFRSF1B | 0.23166026 | 1.61E-08 | positive |
| FBP1 | B2M | 0.23435559 | 1.09E-08 | positive |
| FBP1 | ISG20 | 0.23454443 | 1.06E-08 | positive |
| FBP1 | CCL8 | 0.23461523 | 1.05E-08 | positive |
| FBP1 | VDR | 0.23644778 | 7.97E-09 | positive |
| FBP1 | VAV1 | 0.24314497 | 2.89E-09 | positive |
| FBP1 | HLA-C | 0.24443837 | 2.37E-09 | positive |
| FBP1 | SOCS1 | 0.24474072 | 2.26E-09 | positive |
| FBP1 | CXCL13 | 0.24544608 | 2.03E-09 | positive |
| FBP1 | CTSS | 0.24593705 | 1.88E-09 | positive |
| FBP1 | CIITA | 0.24648848 | 1.73E-09 | positive |
| FBP1 | HLA-B | 0.24991973 | 1.01E-09 | positive |
| FBP1 | LCK | 0.25071725 | 8.88E-10 | positive |
| FBP1 | CSF2RB | 0.25158572 | 7.73E-10 | positive |
| FBP1 | GNLY | 0.25361212 | 5.59E-10 | positive |
| FBP1 | IL15RA | 0.25458872 | 4.78E-10 | positive |
| FBP1 | ARRB1 | 0.25469626 | 4.69E-10 | positive |
| FBP1 | PLAUR | 0.25819359 | 2.66E-10 | positive |
| FBP1 | CCR1 | 0.26003718 | 1.96E-10 | positive |
| FBP1 | MMP12 | 0.2634767 | 1.11E-10 | positive |
| FBP1 | CCR5 | 0.26371255 | 1.06E-10 | positive |
| FBP1 | PTPRC | 0.2646116 | 9.15E-11 | positive |
| FBP1 | CXCR6 | 0.26672324 | 6.40E-11 | positive |
| FBP1 | TAP1 | 0.26727028 | 5.84E-11 | positive |
| FBP1 | TNFRSF18 | 0.26854044 | 4.70E-11 | positive |
| FBP1 | TNFRSF17 | 0.26855879 | 4.68E-11 | positive |
| FBP1 | IL10RA | 0.26894571 | 4.38E-11 | positive |
| FBP1 | C3AR1 | 0.26988622 | 3.73E-11 | positive |
| FBP1 | PTAFR | 0.27092131 | 3.12E-11 | positive |
| FBP1 | TLR2 | 0.27478044 | 1.59E-11 | positive |
| FBP1 | CD74 | 0.27540101 | 1.43E-11 | positive |
| FBP1 | FCGR3A | 0.27690083 | 1.10E-11 | positive |
| FBP1 | OLR1 | 0.27741957 | 1.00E-11 | positive |
| FBP1 | CD4 | 0.27823975 | 8.64E-12 | positive |
| FBP1 | ITGAL | 0.27902958 | 7.51E-12 | positive |
| FBP1 | CSF2RA | 0.2803526 | 5.92E-12 | positive |
| FBP1 | HLA-A | 0.28062887 | 5.63E-12 | positive |
| FBP1 | HLA-DMB | 0.28084812 | 5.42E-12 | positive |
| FBP1 | CD48 | 0.28209919 | 4.32E-12 | positive |
| FBP1 | HLA-DRB5 | 0.28400091 | 3.06E-12 | positive |
| FBP1 | NR1H3 | 0.28735778 | 1.65E-12 | positive |
| FBP1 | TNFSF12 | 0.28829629 | 1.39E-12 | positive |
| FBP1 | CYBB | 0.28970465 | 1.07E-12 | positive |
| FBP1 | HLA-F | 0.29010155 | 9.93E-13 | positive |
| FBP1 | HLA-DRB1 | 0.29101544 | 8.37E-13 | positive |
| FBP1 | NFATC1 | 0.29303228 | 5.73E-13 | positive |
| FBP1 | CD79A | 0.29766434 | 2.37E-13 | positive |
| FBP1 | CXCL9 | 0.30857609 | 2.78E-14 | positive |
| FBP1 | CCL4 | 0.30943307 | 2.34E-14 | positive |
| FBP1 | LCP2 | 0.31461745 | 8.15E-15 | positive |
| FBP1 | IL32 | 0.31470898 | 8.00E-15 | positive |
| FBP1 | CD86 | 0.31678854 | 5.21E-15 | positive |
| FBP1 | ADM | 0.31789096 | 4.14E-15 | positive |
| FBP1 | CCL5 | 0.32351214 | 1.27E-15 | positive |
| FBP1 | CXCR3 | 0.32380892 | 1.20E-15 | positive |
| FBP1 | PRDX1 | 0.32659429 | 6.59E-16 | positive |
| FBP1 | SLC11A1 | 0.3305131 | 2.83E-16 | positive |
| FBP1 | CD3E | 0.33351031 | 1.47E-16 | positive |
| FBP1 | FPR1 | 0.33718688 | 6.49E-17 | positive |
| FBP1 | PDCD1 | 0.33749538 | 6.06E-17 | positive |
| FBP1 | CD14 | 0.3403343 | 3.20E-17 | positive |
| FBP1 | HLA-DMA | 0.34074189 | 2.92E-17 | positive |
| FBP1 | HLA-DPA1 | 0.34176372 | 2.32E-17 | positive |
| FBP1 | HCK | 0.34549686 | 9.88E-18 | positive |
| FBP1 | HLA-DRA | 0.34880106 | 4.60E-18 | positive |
| FBP1 | RNASE2 | 0.35158986 | 2.40E-18 | positive |
| FBP1 | CD3D | 0.35432383 | 1.26E-18 | positive |
| FBP1 | IL2RG | 0.3560628 | 8.30E-19 | positive |
| FBP1 | GMFG | 0.35621722 | 8.00E-19 | positive |
| FBP1 | HLA-DQB1 | 0.37178841 | 1.75E-20 | positive |
| FBP1 | PRF1 | 0.37341245 | 1.16E-20 | positive |
| FBP1 | ITGB2 | 0.37355142 | 1.12E-20 | positive |
| FBP1 | SPP1 | 0.37840337 | 3.22E-21 | positive |
| FBP1 | TYROBP | 0.38989987 | 1.56E-22 | positive |
| FBP1 | HLA-DPB1 | 0.39470356 | 4.24E-23 | positive |
| FBP1 | HLA-DQA1 | 0.39564098 | 3.28E-23 | positive |
| FBP1 | HCST | 0.40795068 | 1.05E-24 | positive |
| FBP1 | CCL17 | 0.40991752 | 5.96E-25 | positive |
| FBP1 | TYMP | 0.41038485 | 5.21E-25 | positive |
| FBP1 | RAC2 | 0.434199 | 4.11E-28 | positive |
| FBP1 | CHIT1 | 0.43708452 | 1.66E-28 | positive |
| FBP1 | LYZ | 0.43868584 | 1.00E-28 | positive |
| FBP1 | FGR | 0.44685141 | 7.29E-30 | positive |
| FBP1 | FCER1G | 0.46810619 | 5.62E-33 | positive |
| FBP1 | FCGR2B | 0.47069419 | 2.27E-33 | positive |
| FBP1 | CCL13 | 0.49163208 | 1.10E-36 | positive |
| FBP1 | GZMB | 0.50624103 | 3.86E-39 | positive |
| FBP1 | CCL7 | 0.51736135 | 4.35E-41 | positive |
| FBP1 | IGHA1 | 0.53122611 | 1.28E-43 | positive |
| FBP1 | IGLV6-57 | 0.55458934 | 3.66E-48 | positive |
| FBP1 | MARCO | 0.55747647 | 9.49E-49 | positive |
| FBP1 | CCL18 | 0.57701716 | 7.11E-53 | positive |
| GMPPB | NR2F1 | -0.219694 | 8.82E-08 | negative |
| GMPPB | MAPK1 | 0.200896 | 1.05E-06 | positive |
| GMPPB | NLRX1 | 0.20238811 | 8.71E-07 | positive |
| GMPPB | TNFRSF1B | 0.20780706 | 4.34E-07 | positive |
| GMPPB | TNFSF13 | 0.21034651 | 3.11E-07 | positive |
| GMPPB | FGR | 0.21362736 | 2.01E-07 | positive |
| GMPPB | SEMA3F | 0.21636305 | 1.39E-07 | positive |
| GMPPB | S100A16 | 0.21861396 | 1.02E-07 | positive |
| GMPPB | ADM2 | 0.22045979 | 7.93E-08 | positive |
| GMPPB | PLXNB1 | 0.22197544 | 6.42E-08 | positive |
| GMPPB | IL4R | 0.22419229 | 4.71E-08 | positive |
| GMPPB | PLXNB2 | 0.23572339 | 8.88E-09 | positive |
| GMPPB | TXLNA | 0.25193478 | 7.31E-10 | positive |
| GMPPB | IL17RC | 0.25426092 | 5.04E-10 | positive |
| GMPPB | SEMA3B | 0.27647197 | 1.18E-11 | positive |
| GMPPB | RHOA | 0.27710857 | 1.06E-11 | positive |
| GMPPB | IKBKE | 0.29237087 | 6.49E-13 | positive |
| GMPPB | SYTL1 | 0.33714556 | 6.55E-17 | positive |
| PSMC4 | ACTA1 | 0.20898726 | 3.72E-07 | positive |
| PSMC4 | TRPC4AP | 0.21003096 | 3.24E-07 | positive |
| PSMC4 | ICAM2 | 0.21240525 | 2.37E-07 | positive |
| PSMC4 | BIRC5 | 0.22702791 | 3.15E-08 | positive |
| PSMC4 | ARG2 | 0.22768404 | 2.87E-08 | positive |
| PSMC4 | PPP4C | 0.23815544 | 6.17E-09 | positive |
| PSMC4 | PGF | 0.23948336 | 5.06E-09 | positive |
| PSMC4 | FABP5 | 0.25356836 | 5.63E-10 | positive |
| PSMC4 | LTBP4 | 0.27123289 | 2.96E-11 | positive |
| PSMC4 | INSL6 | 0.27505217 | 1.52E-11 | positive |
| PSMC4 | PSMD13 | 0.29666505 | 2.87E-13 | positive |
| PSMC4 | BPHL | 0.31892295 | 3.34E-15 | positive |
| PSMC4 | PSMD8 | 0.39009464 | 1.48E-22 | positive |
| PSMC4 | TMSB15A | 0.42306483 | 1.25E-26 | positive |
| PSMC4 | NFKBIB | 0.44299923 | 2.53E-29 | positive |
| PSMC4 | PAK4 | 0.47572922 | 3.80E-34 | positive |
| PSMC4 | AKT2 | 0.71402433 | 1.05E-91 | positive |
| PSMC4 | PSMC4 | 0.95752557 | 6.78E-315 | positive |
